# Risk assessment for perioperative pressure injuries*

**DOI:** 10.1590/1518-8345.2677-3117

**Published:** 2019-01-14

**Authors:** Camila de Assunção Peixoto, Maria Beatriz Guimarães Ferreira, Márcia Marques dos Santos Felix, Patrícia da Silva Pires, Elizabeth Barichello, Maria Helena Barbosa

**Affiliations:** 1 Universidade Federal do Triângulo Mineiro, Instituto de Ciências da Saúde, Uberaba, MG, Brazil.; 2 Prefeitura Municipal de Uberaba, Secretaria Municipal de Saúde, Uberaba, MG, Brazil.

**Keywords:** Pressure Ulcer, Patient Positioning, Perioperative Nursing, Risk Factors, Elective Surgical Procedures, Risk Assessment, Lesão por Pressão, Posicionamento do Paciente, Enfermagem Perioperatória, Fatores de Risco, Procedimentos Cirúrgicos Eletivos, Medição de Risco, Úlcera por Presión, Posicionamiento del Paciente, Enfermería Perioperatoria, Factores de Riesgo, Procedimientos Quirúrgicos Electivos, Medición de Riesgo

## Abstract

**Objectives:**

to evaluate and classify patients according to the Risk Assessment Scale for Perioperative Pressure Injuries; verify the association between sociodemographic and clinical variables and the risk score; and identify the occurrence of pressure injuries due to surgical positioning.

**Method:**

observational, longitudinal, prospective and quantitative study carried out in a teaching hospital with 278 patients submitted to elective surgeries. A sociodemographic and clinical characterization questionnaire and the Risk Assessment Scale for Perioperative Pressure Injuries were used. Descriptive, bivariate and logistic regression analyses were applied.

**Results:**

the majority of patients (56.5%) presented a high risk for perioperative pressure injury. Female sex, elderly group, and altered body mass index values were statistically significant (p < 0.05) for a higher risk of pressure injuries. In 77% of the patients, there were perioperative pressure injuries.

**Conclusion:**

most of the participants presented a high risk for development of perioperative decubitus ulcers. The female sex, elderly group, and altered body mass index were significant factors for increased risk. The Risk Assessment Scale for Perioperative Pressure Injuries allows the early identification of risk of injury, subsidizing the adoption of preventive strategies to ensure the quality of perioperative care.

## RESUMO

**Objetivos:**

avaliar e classificar pacientes segundo a Escala de Avaliação de Risco para Desenvolvimento de Lesões Decorrentes do Posicionamento Cirúrgico; verificar se há associação entre variáveis sociodemográficas, clínicas e escore de risco; e identificar a ocorrência de lesões por pressão, decorrentes do posicionamento cirúrgico.

**Método:**

estudo observacional, longitudinal, prospectivo e quantitativo, realizado em hospital de ensino, com 278 pacientes submetidos a cirurgias eletivas. Utilizaram-se questionário de caracterização sociodemográfica e clínica e Escala de Avaliação de Risco para Desenvolvimento de Lesões Decorrentes do Posicionamento Cirúrgico. Empregaram-se análises descritiva, bivariada e de regressão logística.

**Resultados:**

a maioria dos pacientes (56,5%) apresentou alto risco para lesão perioperatória por posicionamento. Sexo feminino, idoso e valores de índice de massa corporal alterados foram estatisticamente significativos (p<0,05) para maior risco de ocorrência dessas lesões. Em 77% dos pacientes houve lesões por posicionamento.

**Conclusão:**

a maioria dos participantes apresentou alto risco para desenvolvimento de lesão perioperatória por posicionamento. Sexo feminino, idoso e índice de massa corporal alterado foram fatores significativos para aumento do risco. A Escala de Avaliação de Risco para Desenvolvimento de Lesões Decorrentes do Posicionamento Cirúrgico possibilita identificar risco de lesão precocemente, subsidiando a adoção de estratégias preventivas para assegurar a qualidade do cuidado perioperatório.

## RESUMEN

**Objetivos:**

evaluar y clasificar pacientes según la Escala de Evaluación de Riesgo para el Desarrollo de Lesiones Derivadas por Posicionamiento Quirúrgico, verificar si hay asociación entre variables sociodemográficas, clínicas y escore de riesgo e identificar si existen lesiones por presión, derivadas del posicionamiento quirúrgico.

**Método:**

estudio observacional, longitudinal, prospectivo y cuantitativo, realizado en hospital de enseñanza, con 278 pacientes sometidos a cirugías electivas. Se utilizó un cuestionario de caracterización sociodemográfica y clínica y Escala de Evaluación de Riesgo para el Desarrollo de Lesiones Derivadas por Posicionamiento Quirúrgico. Empleamos análisis descriptivos, bivariados y de regresión logística.

**Resultados:**

la mayoría de los pacientes (56,5%) presentó alto riesgo para lesión peri operatoria por posicionamiento. El sexo femenino, ancianos y valores de índice de masa corporal alterados fueron estadísticamente significativos (p <0,05) para mayor riesgo de existencia de esas lesiones. En el 77% de los pacientes hubo lesiones por posicionamiento.

**Conclusión:**

la mayoría de los participantes presentó alto riesgo de desarrollo de lesión peri operatoria por posicionamiento. El sexo femenino, ancianos e índice de masa corporal alterado fueron factores significativos para el aumento del riesgo. La Escala de Evaluación de Riesgo para el Desarrollo de Lesiones Derivadas del Posicionamiento Quirúrgico posibilita identificar precozmente el riesgo de lesión, subsidiando la adopción de estrategias preventivas para asegurar la calidad del periodo perioperatorio.

## Introduction 

Despite technological advances, pressure injuries (PI) caused by surgical positioning still represent a challenge for clinical practice^1^. Because they are considered complications and have a multifactorial etiology, it is difficult to assess the risk of their occurrence in surgical patients^2^, which often compromises the adoption of adequate protective measures for this clientele.

Various incidence rates of perioperative PI are described in the literature. A systematic review of 17 studies published from 2005 to 2011 that evaluated the incidence of these lesions found results ranging from 0.3% to 57.4%^3^.

International researchers also investigated the incidence of perioperative PI derived from surgical positioning and found the following rates: 12.2% in Portugal^4^, 12.7% in Italy^5^ and 13% in the United States of America (USA)^6^.

Surveys in Brazil reported the occurrence of perioperative PI in comparison with other countries: 25% in Paraná^7^, 74% in the Triângulo Mineiro^1^, and 10.1% in São Paulo city^8^.

Effective interventions to prevent skin lesions involve pressure relief during and immediately after the patient lies on the surgical table, on a standard mattress. Examples of more effective devices to prevent this type of injury are: micropulse air mattress, viscoelastic dry polymer mattress cover and gel pads^9^ ^-^ ^10^.

The incidence of these PI varies significantly according to the clinical environment and the individual and clinical characteristics of the patient^11^. The main the extrinsic risk factors are pressure, friction and shear forces, moisture and heat^12^, and the intrinsic factors are age, body weight, nutritional status, presence of comorbidities, immobility or reduced activity levels, fecal incontinence, infection, low hemoglobin level, and surgical risk^9^ ^,^ ^13^ ^-^ ^14^.There are also specific intraoperative factors: prolonged surgical time, surgical positioning, use of anesthetic agents, sedation, vasoconstricting medications, type of surgery, body temperature (hypothermia), type of surgical table mattress, use of devices for positioning, and intraoperative heating and hypotension^13^ ^-^ ^15^.

Despite the existence of high technology preventive devices and the widespread use of the Braden scale in clinical nursing practice, gaps remain on the identification of factors critical to the occurrence of perioperative PI.

In this scenario, given the scarcity of intraoperative risk assessment scales of decubitus ulcers and the need to recognize the risks for elaborating individualized care plans that guarantee safe and quality perioperative care, the application of the Risk Assessment Scale for Perioperative Pressure Injuries (ELPO), a valid and reliable instrument, is recommendable^16^.

The ELPO, developed and validated in Brazil, evaluates the risk of developing injuries resulting from surgical positioning. The score ranges from 7 to 35 points: the higher the score, the greater is the risk of the patient developing pressure injuries. The scale is based on recent evidence and includes factors recommended by scholars^16^.

In addition to ELPO, the Munro Pressure Ulcer Risk Assessment Scale for Perioperative Patients ^17^ and the Scott Triggers Risk Assessment Tool^18^, both included in the recommendations for prevention of perioperative PI of the United States Association of periOperative Registered Nurses (AORN). The Munro Scale evaluates risk factors present in the different operative moments, namely: preoperative, mobility and body mass index (BMI); intraoperative, physical status classification according to the American Society of Anesthesiologists (ASA) scale, and body temperature; and postoperative, duration of the anesthetic-surgical procedure and occurrence of hemorrhage (17). The Scott Triggers tool evaluates the patient’s age, albumin or BMI values, ASA classification, and estimated duration of the surgery^18^.

It is understood that the knowledge of possible contributing factors could support the planning of perioperative nursing care in the process of PI prevention because it would aid to identify patients at risk of developing perioperative PI. In view of this, the following questions were formulated: do patients submitted to elective surgeries have a high ELPO score (score ≥ 20)? Is there any association between sociodemographic variables (sex, age and skin color), clinical variables (BMI, altered hemoglobin values, intraoperative hypothermia), and risk according to the ELPO score? What is the incidence of perioperative PI?

Thus, the purpose of this study was to evaluate and classify patients according to the ELPO score, verify the existence of associations between sociodemographic and clinical variables and the risk score in the ELPO scale, and identify the occurrence of perioperative PI.

## Method

This is an observational, longitudinal, prospective and quantitative study carried out in the surgical center of a large teaching hospital.

Patients aged 18 years and older, of both sexes, undergoing elective surgeries were included in the study. Patients who underwent cardiac surgeries through deliberate hypothermia during the surgical procedure and those who presented at least one of the defining characteristics of Impaired Physical Mobility according to the Nursing Diagnoses Definitions and Classification, which prevented weight and height measurements in the immediate preoperative period, were excluded from the study.

For sampling calculation, the following parameters were adopted: incidence of perioperative PI of 50%, accuracy of 5% and 95% confidence interval, for a finite population of 1000 surgeries, in a total of 278 participants. The recruitment process was non-probabilistic.

For data collection, we used an instrument addressing sociodemographic variables (age, sex and self-reported skin color) and clinic variables (body mass, hemoglobin values, ASA physical status classification, and atrial temperature) of the patient. The Risk Assessment Scale for Perioperative Pressure Injuries (ELPO) is composed of the following variables: duration of the surgery, type of anesthesia, surgical positioning, support surface, positioning of upper and lower limbs, comorbidities and age of the patient^16^.

Prior to data collection, a pilot test was conducted with 12 patients to verify the applicability and suitability of the instrument, but there was no need for alterations. The researchers participated in a training moment for consensus in data collection.

Data collection occurred between February and May 2017, in three moments: preoperative, intraoperative and postoperative. In the immediate preoperative period, sociodemographic variables (age, sex, and skin color) were obtained by means of information provided by the patients at the time of admission to the hospital. Hemoglobin values ​​were consulted in the pre-anesthetic evaluation card or on the Web system of the laboratory of the hospital that was the field of this study. The variable presence of comorbidities was obtained through a verbal report of the patient and confirmation in the physical record. The weight and height of the patient were also collected by means of a digital scale and a vertical stadiometer (adult type Filizola®, previously calibrated) to calculate the BMI.

For nutritional classification in adults, the parameters recommended by the World Health Organization (WHO) were: low weight (BMI < 18.5 kg/m^2^), eutrophic (BMI ≥18.5 and < 25 kg/m^2^), overweight ≥ 25 and < 30 kg/m^2^) and obesity (BMI ≥ 30 kg/m^2^). The Lipschitz classification was adopted for the elderly: low weight for BMI < 22 kg/m^2^, eutrophy for BMI 22-27 kg/m^2^, and obesity for BMI > 27 kg/m^2(^ ^19^. The adoption of different parameters for the elderly is justified by the fact that aging brings changes such as decreased stature, accumulation of adipose tissue, reduction of lean body mass, and decreased amount of water in the body, which directly impact their body composition^19^.

In the intraoperative period, the patient was followed from the entrance into the operating room (OR) until his/her transfer to the post-anesthetic recovery room. The ear temperature was measured in the same ear canal (external ear) with a G-TECH Premium^®^ infrared tympanic thermometer at the following moments: patient admission to the operating room, beginning of anesthesia, beginning of the surgery, and every hour after the anesthetic induction until the moment of the patient’s exit from the OR. The information for the ASA physical status classification was extracted from the anesthetic data in the medical record. It should be noted that the ELPO scale was also applied in this moment; that score 20 was considered as a cut-off point to differentiate the patients’ classification. Those with a score ≤ 19 points were classified as having a lower risk for the development of perioperative PI, while patients with a score ≥ 20 were considered to present a higher risk for this event^16^.

Finally, the patient was evaluated by skin inspection and palpation in the immediate postoperative period (T3), at the time of transfer from the surgical table to the stretcher, and in the first (24 hours), second (48 hours) and third (72 hours) day (T4, T5 and T6) after the surgery in the bed of the hospitalization unit. The identified PI were classified according to the National Pressure Ulcer Advisory Panel (NPUAP) practice guidelines^20^.

The NPUAP classifies pressure lesions in stages 1, 2, 3 and 4, unstageable pressure injury, deep tissue pressure injury, medical device related pressure injuries, and to mucous membranes related pressure injuries. PI stage 1 shows intact skin with non-blanchable erythema. PI stage 2 is characterized by Partial-thickness skin loss with exposed dermis. The wound bed is viable, pink or red, moist, and may also present as an intact or ruptured serum-filled blister. In LPP stage 3 there is full-thickness loss of skin, in which granulation tissue and is often present and slough and/or eschar may be visible. The stage 4 pressure lesion is characterized by full-thickness skin and tissue loss with exposed fascia, muscle, tendon, ligament, cartilage or bone, and there is slough and/or devitalized tissue. Unstageable PI shows full-thickness skin and tissue loss in which the extent of tissue damage within the ulcer cannot be confirmed because it is obscured by slough or eschar. Resulting from friction or shearing, deep tissue PI presents intact or non-intact skin, localized dark red, brown or purple, persistent and non-blanchable area or with separation from the epidermis revealing a dark wound bed or blood-filled blister^20^.

The data collected were analyzed using the SPSS (Statistical Package for the Social Sciences) for Windows, version 22. Absolute and percentage frequency distributions were calculated for categorical variables and measures of central tendency (mean and median) and variability (amplitude and standard deviation) for quantitative variables. A bivariate analysis was used to verify the association between sociodemographic, clinical and anesthetic-surgical variables and the risk of developing perioperative PI according to the ELPO scale. The analysis included measures of association in contingency tables (relative risk, odds ratio and respective confidence intervals) followed by logistic regression, adjusting for other potentially relevant variables. The inferential analyses considered a level of significance of 5% (α = 0.05).

This study is part of a larger project approved by the Research Ethics Committee of the Federal University of the Triângulo Mineiro under the Certificate of Presentation for Ethical Assessment (CAAE) 63030316.9.0000.5154 and Opinion number 1.916.567 / 2017.

## Results

**Figure 1:**
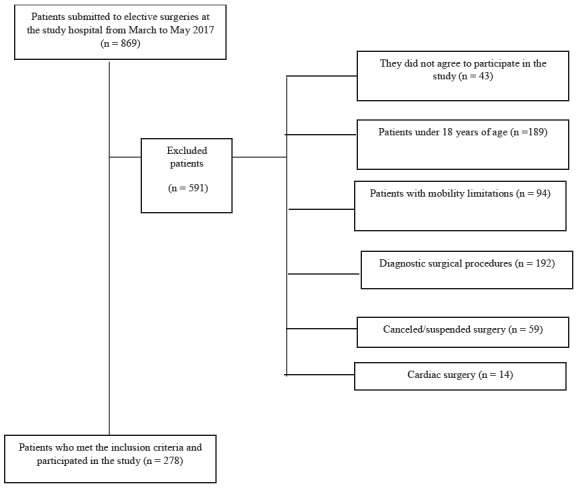
Schematic representation of the selection of patients submitted to elective surgeries (n = 278). Uberaba, MG, Brazil, 2017

Between February and May 2017, a total of 869 patients were submitted to elective surgical interventions at the hospital under investigation. Of these, 278 were included in the study and 591 were excluded, according to Figure 1.

The majority of the participants were female (175; 62.9%), white (162; 58.3%) and adults (203; 73%), and the mean age was 48.7 years, with a minimum of 18 and maximum of 90 years (Table 1).

**Table 1:** Sociodemographic characterization of patients submitted to elective surgeries (n = 278). Uberaba, MG, Brazil, 2017

Variables	n	%
Sex		
Female	175	62.9
Male	103	37.1
Self-reported skin color		
White	162	58.3
Brown	103	37.1
Black	10	3.6
Yellow	1	0.3
Not informed	2	0.7
Age group		
Adults	203	73
Elderly	75	27

Regarding body mass, the mean weight was 73.1 kg (SD = 17.3), with a minimum of 41.6 and maximum of 142.5 kg. The mean height was 1.62 m (SD = 9.3), with a minimum of 1.41 and maximum of 1.88 m. The mean BMI of the participants was 27.7 (SD = 5.9), with a minimum of 17.3 and maximum of 49.1. As for the nutritional classification of the 203 adults, there was a predominance of overweight (71; 25.5%), followed by obesity (62; 22.3%), while among the 75 elderly, 36 (12.9%) were eutrophic.

Only 69 (24.8%) patients presented altered hemoglobin levels, with a mean value of 3.2 g/dl, a minimum of 8 and a maximum of 18 g/dl. Regarding physical status, the majority (158; 56.8%) was classified as ASA II. The mean atrial temperature at the beginning of the anesthetic induction reached 36.4 °C, with a gradual decrease as the anesthetic time increased, so that, after 240 minutes of the onset of anesthesia, it fell to 35.1 °C.

Table 2 shows the results of the ELPO variables adopted in the surgical anesthetic procedures evaluated in the present study.

**Table 2:** Distribution of patients submitted to elective surgeries (n = 278) according to variables present in the Risk Assessment Scale for Perioperative Pressure Injuries (ELPO). Uberaba, MG, Brazil, 2017

Variables	n	%
Type of surgical position		
Supine	102	36.7
Lateral	06	2.2
Trendelenburg	120	43.2
Prone	03	1.1
Lithotomy	47	16.9
Duration of the surgery (hours)		
Up to 1	85	30.6
From 1 to 2	103	37.1
From 2 to 4	80	28.8
From 4 to 6	09	3.2
More than 6	01	0.4
Type of anesthesia		
Local	-	-
Sedation	04	1.4
Regional	119	42.8
General	114	41.0
General + Regional	41	14.7
Support surface		
Viscoelastic mattress + viscoelastic cushions	-	-
Foam mattress + viscoelastic cushions	-	-
Foam mattress + foam cushions	-	-
Foam Mattress + cotton cushions	251	90.3
No use of support surfaces or rigid supports without cushioning or narrow leggings	27	9.7
Limb position		
Anatomic position	15	5.4
Opening of upper limbs < 90°	105	37.8
Knees raised < 90° and opening of lower limbs < 90° or neck without sternal alignment	29	10.4
Knees raised > 90° or opening of lower limbs > 90°	79	28.4
Knees raised > 90° and opening of lower limbs > 90° or opening of upper limbs > 90°	50	18.0
Comorbidities		
No comorbidities	117	42.1
Vascular disease	38	13.7
Diabetes mellitus	16	5.8
Obesity or malnutrition	103	37.1
Pre-diagnosed pressure injury or neuropathy or deep venous thrombosis	04	1.4
Age of the patient		
Between 18 and 39 years	82	29.5
Between 40 and 59 years	121	43.5
Between 60 and 69 years	46	16.5
Between 70 and 79 years	26	9.4
> 80 years	03	1.1

Regarding the type of support surface, cushions in the elbows (right and left) predominated (251; 90.3%), followed by cushions in the occipital region (151; 54.3%) and calves (109; 39.2%).

Regarding the risk for the development of perioperative PI according to the ELPO scale, the majority (157; 56.5%) of the patients evaluated presented a high risk for the event. The mean ELPO score was 20.09 points (SD = 3.63), with a minimum of 13 and a maximum of 29 points.

As for the association between sociodemographic and clinical variables and the ELPO score of the patients submitted to elective surgeries, the female sex, elderly group, and altered BMI were related with a statistically significant greater risk for the development of perioperative PI, with differences (Table 3).

**Table 3:** Bivariate analysis and logistic regression involving the score in the Risk Assessment Scale for Perioperative Pressure Injuries (ELPO*) and clinical and sociodemographic variables of patients submitted to elective surgeries (n = 278). Uberaba, MG, Brazil, 2017

Variables	ELPO* Risk score		RR^†^(CI)^‡^	OR^A§^(IC)^‡^	OR^B׀׀^(IC)^‡^	p^¶^
	High risk	Low risk				
	n (%)	n (%)				
Sex						
Female	104 (59.4)	71 (40.6)	1.155 (0.923 - 1.445)	1.382 (0.846 - 2.256)	2.758 (1.302 - 5.842)	0.008
Male	53 (51.5)	50 (48.5)				
Age group						
Elderly	62 (82.7)	13 (17.3)	1. 766 (1.476 - 2.114)	5.422 (2.807 - 10.473)	14.541 (5.243 - 40.328)	<0.001
Adult	95 (46.8)	108 (53.2)				
Skin color						
White	88 (54.3)	74 (45.7)	0.911 (0.741 - 1.120)	0.804 (0.495 -1.307)	0.966 (0.494 - 1.889)	0.919
Non-white	68 (59.6)	46 (40.4)				
BMI**						
Altered	112 (63.3)	65 (36.7)	1.420 (1.112 - 1.814)	2.144 (1.304 - 3.526)	3.009 (1.466 - 6.177)	0.003
Eutrophic	45 (44.6)	56 (55.4)				
Hypothermia (Taur60°^††^)						
Yes	90 (59.2)	62 (40.8)	0.928 (0.734 - 1.173)	0.284 (0.441 - 1.540)	0.696 (0.340 - 1.426)	0.322
No	37 (63.8)	21 (36.2)				
Hemoglobin						
Altered	40 (58.0)	29 (42.0)	1.036 (0.819 - 1.309)	1.085 (0.625 - 1.881)	1.525 (0.728 - 3.194)	0.264
Normal	117 (56.0)	92 (44.0)				

* Risk Assessment Scale for Perioperative Pressure Injuries; † RR - Relative Risk; ‡ CI - Confidence Interval; § OR^A^ - Crude or non-adjusted odds ratios; || - RC^B^ - Adjusted odds ratios; ¶ significance level (p < 0.05); ** Body mass index; †† Taur60º - Atrial temperature measured after 60 minutes of anesthetic induction

It was observed that 77% (214) of the patients presented pressure injuries due to the surgical position, most of them in stage 1, and only one participant presented stage 2 PI, and another presented deep tissue PI.

## Discussion

The majority of the patients submitted to elective surgeries included in this study were white. The structure of the skin varies between the different colors; in the black race the structure of the stratum corneum is more compact, providing greater resistance to the skin in the face of chemical irritations and/or trauma. The white skin, in turn, is more vulnerable to the occurrence of pressure injuries^21^.

Studies have demonstrated that the nutritional status indicated by albumin levels ≤ 3 g/dL and changes in BMI (low weight, overweight or obesity) may also influence the occurrence of perioperative PI^4^ ^,^ ^7^. In this study, although albumin levels were not assessed, most participants presented changes in BMI.

In the present sample, approximately 25% of the patients had altered hemoglobin levels. Low levels of hemoglobin deserve attention because they imply less transport of nutrients and oxygen to tissues, and consequently become a significant factor involved in the maintenance of skin integrity^22^.

Most of the patients in this study were classified as ASA II with respect to physical status, corroborating the results of another investigation, whose participants classified as ASA II and III presented higher risk and incidence of perioperative PI when compared to those classified as ASA I^4^.

It was found that the atrial temperature decreased gradually as the anesthetic time increased, reaching a mean of 35.1 °C (95.1 °F) 240 minutes after anesthesia. Studies have shown that hypothermia in the intraoperative and postoperative periods occurs in about 60 to 90% of surgical patients and that factors such as anesthetic agents, length of stay in the operating room and duration of the anesthetic-surgical procedure cause a decrease in body temperature^23^ ^-^ ^24^. A decrease 1 °F (0.55 °C) in the body temperature implies an increase of in 20.2% in the risk of development of perioperative PI ^25^.

One of the most significant risk factors for the occurrence of perioperative PI is the duration of the anesthetic-surgical procedure because long periods of immobilization and exposure to pressure cause anoxia, tissue necrosis and consequent skin injury^2^ ^,^ ^13^. One hour of surgery is capable of increasing the patient’s risk for developing this type of injury by 1.07^26^. Surgeries that exceed 2 hours can affect the oxygenation of compressed tissues, favoring the occurrence of PI^27^.

Another important risk factor in the intraoperative phase is the type of anesthesia. This aspect influences the degree of nervous system depression, pain receptors depression, and relaxation of muscles, so that the patient’s defense mechanisms do not offer protection against pressure, leading to susceptibility to pressure injury and pain^9^.

Several surgical positions were analyzed in this study. The Trendelenburg, supine and lithotomy type were the more frequent. Of the several positions and their variations frequently used in anesthetic-surgical procedures, lithotomic position is the one that offers the greater risk of complications. In the supine position, complications only occur in cases where the patient is inadequately positioned and/or when the patient remains in this position for an extended time, favoring the increase of pressure points against the surgical table^28^.

The correct and safe positioning of the patient implies the use of supports and cushions, soft bandages, lowering of the height during the raising of the legs and, especially, adequate support surfaces (SS)^9^.

SS are specialized devices, overlays, mattresses or integrated systems manufactured for pressure redistribution, control of shear or frictional forces on tissue, microclimate maintenance or other therapeutic functions. They should be chosen according to the specific needs of the patient and the type of surgery^29^.

Studies have shown that the non-use of support surfaces during the intraoperative period increases the risk of perioperative PI^16^ ^,^ ^30^. However, the literature reports that support surfaces are little used in surgical patients because of the political, economic and social issues faced in the country, that also affect the health are, do not allow the availability of this resources in many public services, with a direct interference in the prevention of PI^9^.

Some of the objectives of nurses in the intraoperative period involve reduction, relief and redistribution of pressure. These are the three guiding principles to minimize the risk of perioperative PI. Nurses may implement them by using support surfaces to alleviate the pressure as much as possible, considering the specific needs of each patient^31^. It should be emphasized that sheets and blankets should not be used in the positioning of the patient because they decrease the effectiveness of the support surfaces and may actually increase the pressure^15^.

Regarding the presence of comorbidities, diabetes mellitus is considered one risk factor for the occurrence of perioperative PI because its pathophysiology includes a decrease in blood flow that causes tissue perfusion impairment and healing problems due to the difficulty to replace endothelial cells^6^ ^,^ ^10^.

A longitudinal study of patients undergoing major surgeries in northern Italy showed that diabetes mellitus as well as cardiac and vascular diseases are significant risk factors for the development of decubitus ulcers^5^. Another study, developed in an American hospital, showed that patients with a history of diabetes mellitus are more likely to develop pressure injury than those without this comorbidity, with a 49% increased risk^26^.

The early identification of perioperative PI risk through the use of risk assessment scales such as ELPO^16^ is an important step to prevent this complication, since several factors may contribute to its occurrence^13^. Perioperative PI risk is a frequent nursing diagnosis in the Surgical Center and, depending on the surgery type, it can be observed in 100% of the patients^10^.

The present study showed that 56.5% of the patients presented a perioperative PI risk, while another study a majority (53.2%) of participants with ELPO score ≤ 19 points, that is, a lower risk for this type of injury^16^. It is emphasized that an increase of one point in in the scale indicates a 44% higher probability of developing PI^16^.

This study revealed that the variables female gender, elderly group, and altered BMI presented statistically significant results, that is, they were significant contributing factors for a greater risk of perioperative PI. Another study identified a higher perioperative PI rate among men than among women^25^. On the other hand, studies point out that gender is not a significant independent factor for higher PI risk, but it is part of a set of factors that increase the risk of developing these injuries^32^ ^-^ ^33^.

Regarding the association of age group with risk of developing perioperative PI, the literature is in line with the present findings in the sense that this group being the one under highest risk for the development of PI. Researchers have shown that the elderly are the group at higher risk because their skin goes through a physiological process inherent to aging that causes reduction of skin elasticity and texture, muscle mass, inflammatory response, albumin levels, and subcutaneous tissue, making the skin more susceptible to pressure and, consequently, to the development of tissue damage^4^ ^,^ ^32^.

Perioperative complications increase with age. The elderly are, therefore, more exposed to the risk of perioperative PI^16^. A study carried out in a private hospital in the city of São Paulo, Brazil, found that, advancing age was positively related to the occurrence of perioperative PI, with a higher incidence in patients aged 65 years or older (16; 40.0%)^6^.

In contrast to these results, other studies showed that elderly patients did not present a higher risk of developing perioperative PI when compared to adults^4^ ^,^ ^26^.

Regarding the nutritional status, a study corroborated the results of the present research in the sense that BMI was associated with greater risk for the development perioperative PI. In the said study, BMI > 30 Kg/m^2^ was a predisposing factor for the occurrence of PI (p < 0.001)^4^. In turn, another study showed that PI risk was higher in cases of extreme BMI, and lower in eutrophic individuals^34^.

Researchers from a recent literature review found that overweight and low weight increased the perioperative PI risk^10^. Obesity is considered a risk factor for the occurrence of perioperative PI. This happens because more adipose mass can compress blood vessels and dependent nervous structures, reducing tissue perfusion and conducing to injuries^4^. Low weight, on the other hand, can lead to a marked exposure in the patient’s bony prominences, leaving these points more susceptible to the appearance of PI^15^.

The incidence of perioperative PI deserves to be mentioned in this study. It is noteworthy that 77% of the patients submitted to selective surgeries developed this type of lesion at one of the operative moments evaluated. A study of a cohort of 3225 patients submitted to surgical interventions found that 383 (12%) of these people had this type of lesion^26^.

It is important to understand that the incidence of these injuries remains high due to the absence of preventive measures. Moreover, non-compliance or non-observation of norms and/or clinical guidelines and protocols is the main contributory factor^9^.

Due to the variety of surgeries and the peculiarities of each patient, nurses are responsible for assessing the risks to which individuals are exposed in the preoperative phase, as well as the tools and devices available for the implementation of safe and effective actions to prevent complications^13^.

Developing a strategic plan to address risk factors throughout the perioperative period by determining the causes of injury, identifying any barrier that compromises patient safety, and investigating possible interventions that reduce the incidence of this complication may be the key to prevent PI^35^.

A limiting factor in this study was the non-evaluation of the microclimate (heat and moisture of the skin) and the non-follow-up of patients in the postoperative period. However, this did not compromise the reliability of the results. Another limiting factor was the design of the study; descriptive studies do not allow the establishment of cause-and-effect relationship.

This research contributes to the construction of knowledge about the nursing practice in the care of patients in the perioperative period of elective surgeries. Factors that contribute to the greater risk of developing perioperative PI were highlighted. Inserting nurses in care improvement processes is essential because these professionals play a key role in the prevention of perioperative complications. The ELPO scale is a management tool for the clinical practice of nurses and its application can improve the quality of care, patient safety, the evidence-based decision-making process of nurses, and the reduction of pressure injuries arising from surgical positioning.

## Conclusion

The results of this study showed that the majority of the participants were female, white, adult, overweight, with normal hemoglobin values and classified as ASA II. Regarding intraoperative aspects, most surgeries lasted from one to two hours, and regional anesthesia and Trendelenburg position were the most adopted. Intraoperative hypothermia was observed in 82.4% of the patients. The most used support surface was the surgical table with foam mattress and cotton cushions.

Regarding the risk for the development of perioperative PI, the majority of patients presented a high risk. Besides the factors present in the ELPO scale, the variables female sex, elderly group, and altered BMI were statistically significant and represented important risk factors for the occurrence of perioperative PI. Finally, regarding the occurrence of injuries, most of the participants presented perioperative PI. As these injuries are avoidable complications, the importance of quality work on the part of professionals of the perioperative team in the prevention of these lesions stands out.

The present study contributed with the provision of important evidence on the risk of perioperative PI. However, for the generalization of these results, further research is necessary. Additionally to the variables present in the ELPO scale, the correlation with other factors eventually associated with the occurrence of pressure perioperative injuries such as albumin and blood pressure levels has to be investigated. It is suggested that a longitudinal study with an extended follow-up be performed with patients in the postoperative period.
